# Depressive Symptoms and Vegetarian Diets: Results from the Constances Cohort

**DOI:** 10.3390/nu10111695

**Published:** 2018-11-06

**Authors:** Joane Matta, Sébastien Czernichow, Emmanuelle Kesse-Guyot, Nicolas Hoertel, Frédéric Limosin, Marcel Goldberg, Marie Zins, Cedric Lemogne

**Affiliations:** 1 Faculté de Médecine, Sorbonne Paris Cité, Université Paris Descartes, 75006 Paris, France; sebastien.czernichow@aphp.fr (S.C.); nicolas.hoertel@aphp.fr (N.H.); frederic.limosin@aphp.fr (F.L.); marie.zins@inserm.fr (M.Z.); cedric.lemogne@aphp.fr (C.L.); 2 AP-HP, Hôpitaux Universitaires Paris Ouest, Service de Psychiatrie de l’adulte et du sujet âgé, 75015 Paris, France; 3 Inserm, U894, Centre Psychiatrie et Neurosciences, 75014 Paris, France; 4 AP-HP, Nutrition Department, Georges Pompidou european Hospital, Centre Spécialisé Obésité IdF, 75015 Paris, France; 5 Inserm, UMR 1153 Epidemiology and Biostatistics Sorbonne Paris Cité Centre (CRESS), METHODS Team, 75004 Paris, France; 6 Inserm (U1153), Inra, Cnam, University of Paris 5, 7 & 13, 75006 Paris, France; e.kesse@eren.smbh.univ-paris13.fr; 7 Inserm, Population-based Epidemiological Cohorts Unit, UMS 011, 94800 Villejuif, France; Marcel.goldberg@inserm.fr

**Keywords:** depressive symptoms, vegetarian diet, chronic disease

## Abstract

The association between depressive symptoms and vegetarian diets is controversial. This study examines the cross-sectional association between depressive symptoms and vegetarian diets while controlling for potential confounders. Among 90,380 subjects from the population-based Constances cohort, depressive symptoms were defined by a score ≥19 on the Centre of Epidemiologic Studies-Depression (CES-D) scale and diet types (omnivorous, pesco-vegetarian, lacto-ovo-vegetarian and vegan) were determined with a food frequency questionnaire. Associations between depressive symptoms and diet were estimated through logistic regressions adjusting for socio-demographics, other foods, alcohol and tobacco consumption, physical activity and health-related concerns; specificity analyses considered the exclusion of any other food group. Depressive symptoms were associated with pesco-vegetarian and lacto-ovo-vegetarian diets in multivariable analyses (Odds-Ratio [95% confidence interval]: 1.43 [1.19–1.72] and 1.36 [1.09–1.70], respectively), especially in case of low legumes intake (*p* for interaction < 0.0001), as well as with the exclusion of any food group (e.g., 1.37 [1.24–1.52], 1.40 [1.31–1.50], 1.71 [1.49–1.97] for meat, fish and vegetables exclusion, respectively). Regardless of food type, the Odds-Ratio of depressive symptoms gradually increased with the number of excluded food groups (*p* for trend < 0.0001). Depressive symptoms are associated with the exclusion of any food group from the diet, including but not restricted to animal products.

## 1. Introduction

Vegetarian diets have been shown to be associated with a lower risk of cardiovascular diseases and metabolic syndrome [1], as well as some cancers, suggesting that they may be promoted to reduce these major causes of disability and mortality worldwide [2]. However, recent studies have emerged on the possibility of an association between vegetarian diets and mental disorders [3]. In a recent cross-sectional study conducted on a large sample of men from the Avon Longitudinal Study of Parents and Children, vegetarian men had more depressive symptoms after adjustment for several possible confounding factors such as family history of depression, education level, age, ethnic origins and alcohol and tobacco consumption [3]. In another cross-sectional study among Norwegian and Swedish students (mean age 15 years), individuals who consumed less meat were more likely to be depressed after adjustment for physical characteristics, health, family situation, social status, exercise and alcohol and tobacco consumption [4]. Moreover, in Germany, depressive disorders, anxiety disorders and somatoform disorders were more common among vegan adults after adjustment for socio-demographic characteristics with cross-sectional data [5]. However, other large-scale studies did not find an association between vegetarian diets and depressive symptoms. For instance, a cross-sectional study conducted on the religious community Seventh Day Adventist, showed that the adoption of a vegetarian diet was not associated with a negative mood or a higher risk of depressive symptoms [6]. Besides, on a survey on mood, diet and lifestyle factors, an adherence to a strict plant-diet did not negatively affect mood and was even associated with more positive mental outcomes such as less stress and anxiety [7].

These conflicting results may stem from the fact that causal studies are missing and that there is a possible chance of residual confounding since several potentially important confounding factors are not taken into consideration. For instance, the motivations for excluding animal products from diet are diverse [8] and may include health-related concerns. Therefore, one may posit that the association between the adoption of a vegetarian diet and depressive symptoms might be related to the presence of an underlying chronic condition (e.g., diabetes) leading some individuals to make lifestyle modifications with the belief that such diets would enhance their health or prevent the onset of other diseases. In addition, some explanatory mechanisms that have been put forward in studies showing an association between depressive symptoms and vegetarian diets, such as deficiency of vitamin B12 as an example, could not be tested in these studies and, apart from gender differences, it remains unknown whether this association could differ according to other individual characteristics (e.g., socioeconomic status) or other food groups’ consumption. Finally, the specificity of this association regarding the exclusion of animal products versus the exclusion of other food groups was indeed not tested though such knowledge may inform hypotheses on potential mechanisms.

Our main objective was to assess the cross-sectional association between depressive symptoms and different types of diets characterized by the exclusion of animal products (i.e., pesco-vegetarian, lacto-ovo-vegetarian and vegan diets), while adjusting for sociodemographic variables and other health behaviours as well as chronic diseases. We hypothesized that the association between the two variables of interest would substantially decrease or even disappear after controlling for possibly health-related confounding factors, especially chronic diseases. In addition, we explored whether the association between vegetarian diets and depression would differ according to individual characteristics and would be specific to the exclusion of animal products or observed for any food item exclusion.

Aims of the study: to assess the association of depressive symptoms with different types of diets excluding animal products as well as with the exclusion of any other food items

## 2. Methods

### 2.1. Population

Constances is a large, population-based, prospective cohort whose recruitment began in 2012 and will continue until the end of 2018 (expected total size is 200,000 subjects), including volunteers aged 18–69 at baseline and living in 21 selected departments (administrative divisions) throughout metropolitan France, in both rural and urban settings [9]. Participants were selected among individuals covered by the general insurance scheme or partner health mutual societies (in all, 85% of the French population) using a random sampling scheme stratified on place of residence, age, gender, occupation and socioeconomic status. Eligible individuals were invited by mail to participate in the study. Volunteers completed a self-administered questionnaire on lifestyle, health status, medical history, socio-professional status and lifetime employment history and attended a Health Screening Centre for a comprehensive evaluation including a physical examination and laboratory tests. The Constances Cohort has received the authorization of the French Data Protection Authority (Commission Nationale de l’Informatique et des Libertés, CNIL) and the institutional review board of the National Institute for Medical Research (Inserm). The study protocol was approved by the appropriate ethics committee. All subjects included in this study gave an informed consent.

**Figure 1:**
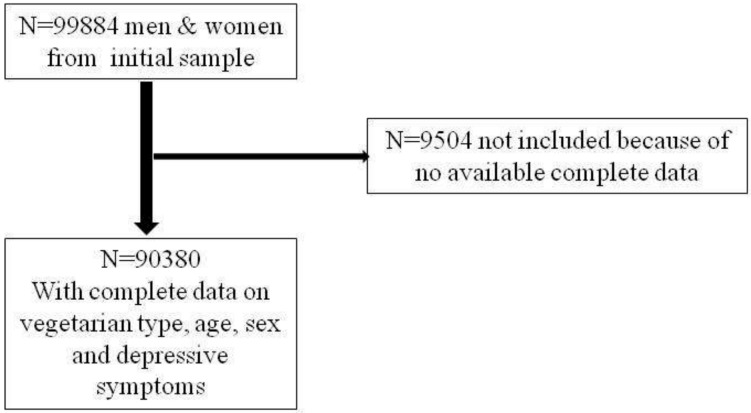
Flowchart of included sample. *n*: number.

The present cross-sectional analyses were restricted to individuals with complete baseline data on vegetarian diet, age, sex and depressive symptoms; data from 90,380 men and women is available (Figure 1).

### 2.2. Variables

#### 2.2.1. Depressive Symptoms

We measured depressive symptoms using the self-administered Centre of Epidemiologic Studies Depression scale (CES-D). This scale evaluates the frequency of depressive symptoms during the previous week (e.g., “I felt depressed”, “I felt everything I did was an effort”, “My sleep was restless”). Responses range from 0 (hardly ever) to 3 (most of the time), resulting in a global score ranging from 0 to 60. Internal consistency of this scale is generally high (α = 0.90 in the Constances cohort). Depression was defined by a CES-D score ≥19, according to the validated cut-off of the French version (sensitivity/specificity for the diagnosis of major depression: 0.85/0.86) [10].

#### 2.2.2. Diet Type

Diet information was obtained using the following nine items from a 24-item qualitative food frequency questionnaire: meat, poultry, fish, eggs, milk and dairies, legumes, fruits, vegetables and grains. Each food item on the questionnaire was measured using the question: “How frequently do you consume this item” on a scale ranging from never or almost never to more than once per day; quantity was not measured. Individuals answering never or almost never were considered as not consuming the food product. For this study, five food items were used in order to create the following groups of diet type: (1) omnivorous if individuals consumed meat, poultry, fish, eggs and milk and dairies; (2) pesco-vegetarian if they did not consume any meat nor poultry but consumed fish, eggs and milk and dairies; (3) lacto-ovo-vegetarian if they consumed eggs and milk and dairies but omitted meat, poultry and fish; and (4) vegan if they did not consume these animal products.

For post hoc stratified analyses, legumes consumption was categorized as: (1) low if they consumed the food item 0–1 time per week; (2) medium if they consumed the food item 2–6 times per week and (3) high if they consumed the food item 1 time per day or more.

#### 2.2.3. Cardio-Metabolic Variables

Fasting blood samples were taken to measure total blood cholesterol, glycaemia and triglyceridemia. Diabetes was defined as a blood glucose level ≥7 mmol/L or reported treatment for diabetes. Dyslipidaemia was defined as reported treatment for high blood cholesterol or triglycerides or a total cholesterol level >6.206 mmol/L, or triglycerides level >2.26 mmol/L [11]. Blood pressure was measured after a 5-min rest period, using an automated oscillometric sphygmomanometer and individuals were considered as having hypertension if they had a systolic or diastolic blood pressure higher than 90/140 mmHg. Weight and height were measured in health centres and BMI (kg/m^2^) was calculated, with obesity being defined as a BMI ≥ 30 kg/m^2^. All anthropometric and blood pressure measurements were obtained following standardized procedures [12,13].

#### 2.2.4. Other Conditions

Cancers (all types) and chronic kidney disease were self-reported through specific questions. Individuals were considered as having anaemia if they had a haemoglobin <130 g/L for men and <120 g/L for women. These conditions were considered because of their potential relationships with plant-based diets, either based on potential consequences (e.g., iron deficiency leading to anaemia), lay beliefs (e.g., that vegetarian diets may help cure cancer) or physicians’ counselling (e.g., a low protein diet in chronic kidney disease).

#### 2.2.5. Physical Activity

Physical activity outside work was determined by a calculated score ranging from 0 (i.e., being very active) to 6 (not active at all). Questions used for the score’s calculation included: (1) in the past 12 months have you regularly engaged in gardening, cleaning or handy work?; (2) in the past 12 months have you regularly practiced any sports (aside from gardening, cleaning or handy work)?; and (3) in the past 12 months have you regularly gone on biking or walking trips (for work or leisure)? The final score is only calculated if the 3 questions are answered, otherwise the data is considered missing.

#### 2.2.6. Other Variables

Education was categorized into four levels: less than or equal to high school diploma, undergraduate degree and postgraduate degree or other. Household monthly income was categorized into three levels: <1500 euros, 1500–2800 euros and ≥2800 euros. Smoking was categorized into never, current or former smokers. Alcohol intake was categorized as 1 time per week or more; 2–3 times per month, 1 time per month or less and never. Self-rated health was determined by the question: “How would you judge the state of your general health?” The participants respond on an 8-point like scale (1 = very good, 8 = very poor). Finally, we also considered as a potential confounding variable the response to a specific question exploring whether the participant considered eating merely as a mean to stay healthy (yes vs. no).

#### 2.2.7. Statistical Analyses

Descriptive statistics were performed to provide characteristics of the sample by diet type and are presented as means ± standard deviations (SD) or percentages as appropriate.

The association between diet type and depressive symptoms was estimated with odds ratios (OR) and their 95% confidence intervals (CI) computed through logistic regression analysis models. At first, four logistic regression models were employed to investigate the association between diet type and depressive symptoms as a binary variable (i.e., CES-D score ≥19 vs. <19). Diet type was considered as a 4-class categorical variable (omnivorous, pesco-vegetarian, lacto-ovo-vegetarian and vegan). These four models were adjusted for potential confounding variables that may explain the association between diet type and depressive symptoms: model 1 represents the crude association; model 2 was adjusted for sociodemographic variables (age, sex, education, household income); model 3 (main model) was further adjusted for frequency of fruits, vegetables, legumes and grains consumption; model 4 was further adjusted for other health behaviours (i.e., smoking, alcohol consumption and physical activity). An additional model 4’ was then computed after exclusion of individuals suffering from at least one of the above-mentioned chronic conditions (as listed in the tables) based on model 3 further adjusted for self-rated health and “eating to stay healthy.” This model 4’ which aimed to challenge the hypothesis of vegetarian diets as being merely a health behaviour, was therefore not adjusted on other health behaviours (i.e., smoking, alcohol consumption and physical activity) to prevent over adjustment.

Further exploratory analyses of interactions between vegetarian diets and sociodemographic factors (age divided into three categories: less than or equal to 35, between 35–60 and 60 or above; sex; education; and income), health behaviours (physical activity, smoking and alcohol intake), the presence of at least one chronic disease and other food groups consumption (fruits, vegetables, legumes and grains) and eating to stay healthy were performed to refine our understanding of the results obtained from the logistic regression analyses. Since we did not have a priori hypotheses for these exploratory analyses, statistical significance was set at a Bonferroni-corrected *p*-value < 0.0001 to reduce the risk of false positives due to multiple testing. Should a significant interaction be found, further stratified analyses were performed.

Finally, to test whether the association between depressive symptoms and vegetarian diet types was specific to the exclusion of animal products, regression analysis was conducted for the association of depressive symptoms and each food item (meat, poultry, fish, eggs, milk and dairies, fruits, vegetables, legumes and grains) as a binary variable (never or almost consumed versus consumed without any emphasis on frequency if consumed) in separate models. Since the results of these last analyses suggest that all food items were associated with depression when excluded, further post hoc analyses were based on the number of excluded food items (none, one, two, three or more) rather than on single food items.

## 3. Results

Table 1 presents the characteristics of the 90,380 study participants by diet type. Among the 99, 884 participants in the initial sample, 9504 were not included because of missing values. Participants with a vegetarian or vegan diet were more likely to be female and younger than those with an omnivorous diet. The unadjusted prevalence of depressive symptoms was highest in the vegan diet group (28.4%) and lowest in the omnivorous diet group (16.2%) when using a CES-D score ≥19.

Table 2 shows the results of logistic regression analyses using depressive symptoms as the dependent variable. The association between depressive symptoms and diet type was significant in model 1 (i.e., crude associations) (*p* < 0.05). Although adjustment for socio-demographic factors resulted in a substantial attenuation (47.9%, 60.0% of OR reduction for pesco-vegetarian and lacto-ovo-vegetarian diets, respectively), this association remained significant after controlling for all potential confounders in model 4 (OR [95% CI]: 1.43 [1.19–1.72] and 1.36 [1.09–1.70], for pesco-vegetarian and lacto-ovo-vegetarian diets, respectively). The removal of individuals with chronic diseases (model 4’) with further adjustment for self-rated health and “eating to stay healthy” did not change the results’ significance.

Exploratory analyses aiming at identifying effect modifiers have found a significant interaction with legumes consumption and “eating to stay healthy” (*p* < 0.0001 and *p* = 0.0002, respectively) (Supplementary Table S1). Further stratifications by legumes consumption showed that vegetarian diets were associated with depressive symptoms mostly in participants with low legumes intake (Supplementary Table S2a) but not in those with high legumes intake. Further stratification according to the “eating to stay healthy” variable (yes vs. no) showed that lacto-ovo-vegetarian and vegan diets were associated with depressive symptoms to a lower extent in participants reporting eating as being merely a mean to stay healthy (Supplementary Table S2b).

Table 3 shows the results of specificity analyses dealing with the association of depressive symptoms with the exclusion of each food item (modelled as a binary variable, never consumed vs. consumed irrespective of the frequency). The exclusion of each food item was associated with depressive symptoms with OR [95% CI] ranging from 1.22 [1.13–1.31] for eggs exclusion to 1.71 [1.49–1.97] for vegetables exclusion. The OR of depressive symptoms gradually increased with the number of excluded food items (*p* for trend < 0.0001 for crude and adjusted model) (Table 4).

## 4. Discussion

### 4.1. Main Findings

We took advantage of the Constances cohort to examine the association between depressive symptoms and vegetarian diets while adjusting for potential confounders and looking for potential effect modifiers. Specifically, we hypothesized that this association would be at least partially explained by health-related concerns. In addition, we sought to examine whether this association would be specific of vegetarian diets compared to other food group exclusions. Our results were not in accordance with our hypothesis and showed associations of depressive symptoms with pesco-vegetarians and lacto-ovo-vegetarians that remained significant even after adjusting for potential confounders or excluding participants with chronic diseases. Furthermore, these associations were indeed of lower magnitude among participants considering eating as a way to stay healthy. In addition, depression was associated with the exclusion of any food group, suggesting that vegetarian diets could represent only a particular instance of a broader phenomenon associated with food exclusion. For instance, vegetable-free diets were similarly associated with depressive symptoms as were meat-free diets. Indeed, depressive symptoms were positively associated with the number of excluded food items, regardless of the type of excluded food items. In contrast, taking into account potential confounding factors, depressive symptoms did not increase from pesco-vegetarian and lacto-ovo-vegetarian diets to the vegan diet, for which no significant association was observed. However, more restrictive vegetarian diets, such as vegan diet, are very specific food restriction patterns, thus possibly driven by special motives that may relate to depression to a lesser extent [8]. The small sample size of this subgroup limits our ability to interpret this lack of statistical significance.

### 4.2. Strengths and Limitations

The strengths of our study include the large sample and the design of the Constances cohort that allowed us to examine different types of vegetarian diets as well as a large scope of potential confounders or effect modifiers. All clinical data have been collected at Health Screening Centres (HSC) in France with routine protocols and check-ups having been conducted by specialized teams [13]. In addition, a field-pilot study that was performed in 2010 in seven HSCs, which included about 3500 subjects, showed a good validity of our collected data [5]. Our study confirms and extends previous results showing a cross-sectional association between depression and vegetarian diets [3,4,14] while further taking into consideration other possible confounders. For instance, to our knowledge, the present study was the first to consider the role of several chronic diseases and to include a measure of haemoglobin to assess anaemia as a potential mediator. Finally, the way we capture vegetarian diets (i.e., through actual food frequency questionnaire) is thought to be more accurate than relying on simple questions based on labels (e.g., “are you vegetarian?”) which may sometimes overestimate the associations [15]. For instance, participants considered as having a vegan diet here were not those who defined themselves as “vegan” but rather those who simply report not eating animal products.

Limitations include the cross-sectional nature of the data which restricts the possibility of drawing causality especially because it is impossible to tell if the diet type preceded or succeeded the depressive symptoms. Further limitations include the absence of some dietary data and in particular the impossibility to calculate energy intake which is an important factor for investigating nutritional issues. We also cannot draw out the possibility of residual confounding: for instance, we lacked an assessment of eating disorders, which could be associated with both depression and food restrictions. Likewise, personality was not assessed though it may affect both variables of interest. For example, vegetarianism may be associated to perfectionism [16], which by itself may possibly reflect vulnerability for depressive symptoms [17]. Although people who are not fluent in French may receive assistance in completing the questionnaire, not being fluent in French might have been a cause of selection bias.

Major depression was not assessed in the present study. Finally, although our main proxy for depression was based on a well-validated measure of depressive symptoms [10], our results may not apply on individuals with major depression but rather on the larger population of those experiencing depressive symptoms.

### 4.3. Explanatory Hypotheses

Several factors may underlie the association of depressive symptoms with the exclusion of one or many food items from the diet. For instance, a deficiency in omega-3 fatty acids because of the exclusion of fish from the diet has been suggested as a potential mediator linking vegetarianism to depression [18]. The present analyses do not support this explanation, since there was no increase in the risk of depression with further restriction of animal products from the diet, that is from the exclusion of meat and chicken to further exclusion of fish and eggs, milk and dairies (OR [95% CI] 0.95 (0.71–1.26) and 0.85 (0.47–1.55) for lacto-ovo-vegetarian and vegan diet, respectively, using pesco-vegetarian diet as a reference in the association between diet type and depressive symptoms and adjusting for all covariates of model 4). Odds of having depressive symptoms were indeed noticed in the pesco-vegetarian diet group, that is, as soon as meat and chicken were removed from the diet.

The association between depressive symptoms and food items exclusion, including but not restricted to animal products, could also be explained by the deficiency in iron, vitamin B12, calcium, zinc and vitamin D [19,20]. For instance, the intake of vitamin B12 could be inadequate in a vegan diet without supplementation. Interestingly, as shown by an interaction surviving a Bonferroni-corrected statistical threshold, the association between vegetarian diets and depression dramatically changed according to the levels of legumes consumption. Specifically, this association decreased as legumes intake increased. Since legumes intake is the most available source of proteins-rich foods beside animal products, it may capture the eating behaviour of the most health-conscious vegetarians, thus less prone to deficiencies. This hypothesis would also be consistent with the smaller association of vegetarian diet with depression among participants considering eating as a way to stay healthy. Indeed, as stated by the American Dietetic Association, appropriately planned vegetarian diets, including vegan diets, are nutritionally adequate [21]. Several other results from the present study are not consistent with the hypothesis of nutrients deficiency as a potential mediator between vegetarian diets and depressive symptoms. First, given that anaemia is associated with depressive symptoms [22] and may result from vitamin B12 or iron deficiency, it may explain the association between depressive symptoms and vegetarianism. Therefore, we have conducted sensitivity analyses and removed individuals with this condition, diagnosed based on haemoglobin levels but this still did not change the significance of the association between depressive symptoms and vegetarianism. Anaemia and thus iron or vitamin B12, are thus unlikely to explain this association.

Although we cannot rule out the mediating role of other potential nutrient deficiencies such as vitamin D [23], reverse causality must also be considered. For instance, a German study found that the adoption of a vegetarian diet seemed to follow the onset of a mental condition [14]. However, although many studies have examined the association between depressive symptoms and diet quality, knowledge about the relationships between depressive symptoms and food exclusion is scarce, especially regarding major depression [24]. It may be surprising given that depressive mood is associated with changes in appetite that feature among the core symptoms of major depression, so that food item exclusion could be considered as potentially signalling depression. These changes in eating behaviour may relate to aberrant functioning of the neural processing of reward and interoceptive signals observed in individuals with major depression [25]. However, the patterns of abnormal eating behaviour may differ across depression subtypes [26]. In addition, comorbid eating disorders may also account to some extent for the association between depressive symptoms and any food item exclusion among some individuals [27,28,29].

Some studies’ results are not in line with our findings, mainly the results obtained from the adults of the Seventh Day Adventist Cohort [6] who seemed to have a better mental profile when adopting a vegetarian diet. This religious community is somewhat different than the Constances cohort in that it has not been drawn from the general population and where vegetarianism is more seen as a support for a religious view rather than a diet approach; the religious view is linked with a sense of belonging to a well-defined community, which is known to protect from depression [30]. In a recent systematic review and meta-analysis regarding depressive symptoms and diet quality, Molendjik et al. [31] also reported that adherence to high-quality, healthy diets including but not restricted to pro-vegetarian diets was associated with lower depression incidence in a linear dose-response pattern [31]. Our results show that the association between depressive symptoms and vegetarian diets is not specific to the exclusion of animal products. Furthermore, this association may depend upon the consumption of other foods and may constitute an impetus to reanalyse older datasets based on data-driven eating patterns rather than isolated food items or theory-driven eating patterns.

## 5. Conclusions

Even though vegetarian diets have been associated with better physical health in several studies, at a population level, they have also been associated with depressive symptoms. However, the fact that any food item exclusion was associated with increased risk of depression suggests that the association between vegetarian diets and depression could be only a particular instance of a broader association between depressive symptoms and food exclusion, regardless of food types. This may not apply to very specific food restriction patterns such as vegan diet but further studies including a larger sample of participants with a vegan diet are needed. Longitudinal data are also needed in order to examine the temporal direction of this association and to check whether depressive symptoms follow or precede the exclusion of some food items including but not restricted to animal products. The long-term follow-up planned for the Constances study will allow us to answer this question in the near future. Additional potential mediators (e.g., deficiencies beyond those resulting in anaemia) or confounders (e.g., personality, eating disorders) have to be also taken into consideration.

## Figures and Tables

**Table 1:** Participant’s characteristics by diet type.

Diet Type	Omnivorous (*n* = 88,905)	Pesco-vegetarian ^†^ (*n* = 832)	Lacto-ovo-vegetarian ^‡^ (*n* = 562)	Vegan ^§^ (*n* = 81)	*p*
**Age**	47.40 ± 13.66	44.97 ± 13.67	41.48 ± 13.78	37.27 ± 12.70	<0.001
**Sex**					<0.001
Men	41,941 (47.18)	196 (23.56)	179 (31.85)	29 (35.80)	
Women	46,964 (52.82)	636 (76.44)	383 (68.15)	52 (64.20)	
**Depressive symptoms**					<0.001
CES-D score ≥ 19	14,391 (16.19)	212 (25.48)	158 (28.11)	23 (28.40)	
**Marital status**					<0.001
(*n* missing = 1250)					
Married	52,026 (59.34)	297 (36.22)	183 (32.97)	24 (29.63)	
Single	35,648 (40.66)	523 (63.78)	372 (67.03)	57 (70.37)	
**Household income**					<0.001
(*n* missing = 1437)					
<1500 euros or does not want to answer	14,594 (16.68)	227 (27.96)	194 (35.14)	35 (43.21)	
1500 to less than 2800 euros	22,992 (26.28)	256 (31.53)	172 (31.16)	29 (35.80)	
≥2800 euros	49,912 (57.04)	329 (40.52)	186 (33.70)	17 (20.99)	
**Education level**					<0.001
(*n* missing = 761)					
≤high school diploma or other diploma	38,174 (43.30)	275 (33.33)	207 (37.16)	38 (46.91)	
Undergraduate degree	29,945 (33.97)	306 (37.09)	205 (36.80)	19 (23.46)	
Postgraduate degree	20,037 (22.73)	244 (29.58)	145 (26.03)	24 (29.63)	
**Other foods consumption**					
No fruit consumption	3280 (3.71)	25 (3.02)	26 (4.68)	11 (13.75)	<0.0001
(*n* missing = 540)					
No vegetable consumption	1401 (1.59)	16 (1.93)	32 (5.71)	15 (18.52)	<0.0001
(*n* missing = 563)					
No legume consumption	15637 (17.74)	125 (15.15)	106 (18.96)	18 (22.22)	0.140
(*n* missing = 773)					
No grain consumption	602 (0.68)	37 (4.49)	21 (3.76)	9 (11.11)	<0.0001
(*n* missing = 571)					
**Physical activity**					<0.001
(*n* missing = 2470)					
Low	24,142 (27.92)	156 (19.24)	134 (24.50)	20 (25.32)	
Moderate	36,690 (45.90)	392 (48.34)	259 (47.35)	35 (44.30)	
High	22,641 (26.18)	263 (32.43)	154 (28.15)	24 (30.38)	
**Smoking**					0.058
(*n* missing = 3479)					
Non-Smokers	39,929 (46.70)	365 (46.09)	241 (44.80)	42 (55.26)	
Smokers	16,600 (19.42)	136 (17.17)	120 (22.30)	21 (27.63)	
Ex-smokers	28,966 (33.88)	291 (36.74)	177 (32.90)	13 (17.11)	
**Alcohol intake**					<0.001
(*n* missing = 5197)					
1/week or more	52,165 (62.22)	384 (50.66)	213 (41.85)	28 (37.33)	
2–3/months	16,968 (20.24)	158 (20.84)	102 (20.04)	19 (25.33)	
1/month or less	11,222 (13.38)	142 (18.73)	117 (22.99)	16 (21.33)	
Never	3486 (4.16)	74 (9.76)	77 (15.13)	12 (16)	
**Anaemia**	2718 (3.07)	64 (7.71)	34 (6.09)	7 (8.64)	<0.001
(*n* missing = 455)					
**Chronic kidney disease**	316 (0.36)	3 (0.37)	1 (0.18)	1 (1.25)	0.523
(*n* missing = 1819)					
**Diabetes**	3036 (3.49)	11 (1.36)	7 (1.26)	2 (2.47)	0.0002
(*n* missing = 2057)					
**Dyslipidaemia**	27,691 (31.93)	181 (22.35)	103 (18.90)	11 (13.58)	<0.0001
(*n* missing = 2013)					
**Cancer (all types)**	4534 (5.21)	42 (5.16)	13 (2.36)	0	0.0036
(*n* missing = 1992)					
**Obesity**	10,639 (12.10)	42 (5.08)	37 (6.62)	5 (6.33)	<0.0001
(*n* missing = 1015)					
**Hypertension**	25,039 (28.57)	134 (16.40)	86 (15.50)	15 (18.52)	<0.0001
(*n* missing = 1297)					
**Eating to stay healthy**	42,342 (47.63)	469 (56.37)	309 (54.98)	47 (58.02)	<0.0001
**Self-rated health**	2.79 ± 1.21	2.75 ± 1.26	2.75 ± 1.34	2.82 ± 1.43	0.809
(*n* missing = 3326)					

Figures indicate ***n*** (%) except for age and self-rated health where they indicate mean (standard deviation); ^†^ not eating meat or poultry; ^‡^ not eating meat, poultry or fish; ^§^ not eating meat, poultry, fish, eggs or milk and dairies. Other foods consumption (fruits, vegetables, legumes, grains) is presented as never or almost never consumed versus consumed.

**Table 2:** Odds-Ratios (95% confidence interval) for the association of diet type with depressive symptoms in logistic regressions.

Type of Diet	Model 1	Model 2	Model 3	Model 4	Model 4’
Omnivorous	1	1	1	1	1
Pesco-vegetarian ^†^	1.71 (1.43–2.04) *	1.37 (1.15–1.64) *	1.42 (1.18–1.71) *	1.43 (1.19–1.72) *	1.55 (1.22–1.98) *
Lacto-ovo-vegetarian ^‡^	1.85 (1.50–2.28) *	1.34 (1.08–1.66) *	1.40(1.12–1.75) *	1.36 (1.09–1.70) *	1.44 (1.08–1.92) *
Vegan ^§^	1.75 (1.01–3.03) *	1.16 (0.66–2.01)	1.24 (0.70–2.20)	1.23 (0.69–2.17)	1.18 (0.56–2.47)

^†^ not eating meat or poultry; ^‡^ not eating meat, poultry or fish; ^§^ not eating meat, poultry, fish, eggs or milk and dairies; Model 1: diet type; Model 2: Model 1 further adjusting for age, sex, education and income; Model 3: Model 2 further adjusting for fruits, vegetables, legumes and grains consumption; Model 4: Model 3 further adjusting for smoking, alcohol and physical activity; Model 4: Model 3 further adjusting for perceived health and “eating to stay healthy” after removal of individuals with chronic conditions (i.e., diabetes, obesity, hypertension, dyslipidaemia, cancer, anaemia, chronic kidney disease). ***n*** = 77,010 (i.e., participants with no missing data for the variables included in model 4) except for model 4’ (***n*** = 38,096).

**Table 3:** Odds-Ratios (95% confidence interval) for the association of food item exclusion with depressive symptoms in logistic regressions.

	Crude OR (95% CI)	Adjusted OR (95% CI) ^†^
Meat	1.78 (1.62–1.96)	1.37 (1.24–1.52)
Poultry	1.83 (1.64–2.04)	1.50 (1.34–1.68)
Fish	1.82 (1.71–1.94)	1.40 (1.31–1.50)
Eggs	1.43 (1.34-1.54)	1.22 (1.13–1.31)
Milk and dairies	1.32 (1.23–1.42)	1.24 (1.16–1.34)
Fruits	1.99 (1.82–2.18)	1.62 (1.48–1.78)
Vegetables	2.30 (2.02–2.63)	1.71 (1.49–1.97)
Legumes	1.42 (1.35–1.49)	1.22 (1.16–1.29)
Grains	1.96 (1.61–2.39)	1.61 (1.31–1.97)

Models included only one food item (never or almost never consumed versus consumed) ^†^ adjusted for age, sex, income, education, alcohol intake, smoking and physical activity. ***n*** = 74,655 (i.e., participants with no missing data for the variables included in the adjusted models).

**Table 4:** Odds-Ratios (95% confidence interval) for the association of the number of excluded food items with depressive symptoms.

	Crude OR (95% CI)	Adjusted OR (95% CI) ^†^
No exclusion	1	1
Exclusion of 1 food item	1.34 (1.27–1.40)	1.20 (1.14–1.26)
Exclusion of 2 food items	1.74 (1.62–1.87)	1.40 (1.30–1.50)
Exclusion of more than 3 food items	3.32 (2.89–3.81)	2.25 (1.95–2.60)

^†^ adjusted for age, sex, income, education, alcohol intake, smoking and physical activity. ***n*** = 73,348 (i.e., participants with no missing data for the variables included in the adjusted model).

## Supplementary Materials

The following are available online at http://www.mdpi.com/2072-6643/10/11/1695/s1, Table S1: Interaction of diet type with each variable in logistic regressions predicting depressive symptoms, Table S2a: Crude Odds-Ratios (95% confidence interval) for the association of diet type with depressive symptoms in logistic regressions according to legumes intake, Table S2b: Crude Odds-Ratios (95% confidence interval) for the association of diet type with depressive symptoms in logistic regressions according to the variable ‘eating to stay healthy’ (yes versus no).
