# Supplementary material for: Depressive Symptoms and Vegetarian Diets: Results from the Constances Cohort
Source: Nutrients. 2018 Nov 6;10(11):1695. doi: 10.3390/nu10111695 (PMC6267287; doi:10.3390/nu10111695)
Supplement: Supplementary file 1 [file nutrients-10-01695-s001.zip › supplementary files/supplementary table 1.docx]

**Supplementary table 1: Interaction of diet type with each variable in logistic regressions predicting depressive symptoms**

| **Separate models with separate interaction terms** | **OR (95% CI)** | **P** |
| --- | --- | --- |
| Age by diet type | 1.00 (1.00-1.01) | 0.003 |
| Sex by diet type | 0.93 (0.79-1.09) | 0.36 |
| Education by diet type | 1.01 (0.93-1.09) | 0.69 |
| Household income by diet type | 0.99 (0.91-1.07) | 0.83 |
| Fruit intake by diet type | 0.98 (0.90-1.06) | 0.62 |
| Vegetable intake by diet type | 0.95 (0.87-1.03) | 0.24 |
| Legumes intake by diet type | 0.69 (0.61-0.78) | **<0.0001** |
| Grains intake by diet type | 0.88 (0.64-1.20) | 0.41 |
| Physical activity by diet type | 1.03 (0.93-1.13) | 0.48 |
| Smoking by diet type | 1.02 (0.94-1.10) | 0.53 |
| Alcohol intake by diet type | 0.91 (0.86-0.97) | 0.01 |
| Perceived health by diet type | 0.99 (0.94-1.06) | 0.98 |
| Diabetes by diet type | 1.33 (0.77-2.29) | 0.28 |
| Obesity by diet type | 1.23 (0.95-1.59) | 0.12 |
| Hypertension by diet type | 1.25 (1.05-1.50) | 0.01 |
| Chronic kidney disease by diet type | 2.88 (0.64-12.93) | 0.15 |
| Anemia by diet type | 0.94 (0.72-1.22) | 0.62 |
| Cancer by diet type | 0.60 (0.33-1.07) | 0.08 |
| Eating to stay healthy by diet type | 0.76 (0.66-0.87) | **0.0002** |

Each model included diet type, the potential effect modifier and the interaction of both.

Statistical significance level was set at a Bonferroni corrected P-value <0.0001
