# Supplementary material for: Depressive Symptoms and Vegetarian Diets: Results from the Constances Cohort
Source: Nutrients. 2018 Nov 6;10(11):1695. doi: 10.3390/nu10111695 (PMC6267287; doi:10.3390/nu10111695)
Supplement: Supplementary file 1 [file nutrients-10-01695-s001.zip › supplementary files/Supplementary table 2a.docx]

**Supplementary table 2a: Crude Odds-Ratios (95% confidence interval) for the association of diet type with depressive symptoms in logistic regressions according to legumes intake**

| **Legumes: low intake** | OR (95% CI) |
| --- | --- |
| Omnivorous diet (74890; 85.0% of this group) | 1 |
| Pesco-vegetarian diet^†^ (520; 63.0% of this group) | 2.00 (1.63-2.43) |
| Lacto-ovo-vegetarian / vegan diet^‡^ (332; 51.6% of this group) | 2.84 (2.27-3.57) |
| **Legumes: medium intake** |  |
| Omnivorous diet (12220; 13.9% of this group) | 1 |
| Pesco-vegetarian diet^†^ (259; 31.4% of this group) | 1.51 (1.12-2.03) |
| Lacto-ovo-vegetarian / vegan diet^‡^ (258; 40.1% of this group) | 1.38 (1.02-1.87) |
| **Legumes: high intake** |  |
| Omnivorous diet (1032; 1.2% of this group) | 1 |
| Pesco-vegetarian diet^†^ (46; 5.6% of this group) | 0.60 (0.26-1.37) |
| Lacto-ovo-vegetarian / vegan diet^b^ (50; 7.8% of this group) | 0.64 (0.29-1.38) |

^†^ not eating meat or poultry

^‡^ not eating meat, poultry or fish (lacto-ovo-vegetarian and vegan diets were combined because of the low number of participants with vegan diet in the stratification analyses)
