# Supplementary material for: Depressive Symptoms and Vegetarian Diets: Results from the Constances Cohort
Source: Nutrients. 2018 Nov 6;10(11):1695. doi: 10.3390/nu10111695 (PMC6267287; doi:10.3390/nu10111695)
Supplement: Supplementary file 1 [file nutrients-10-01695-s001.zip › supplementary files/Supplementary table 2b.docx]

**Supplementary table 2b: Crude Odds-Ratios (95% confidence interval) for the association of diet type with depressive symptoms in logistic regressions according to the variable ‘eating to stay healthy’ (yes *versus* no)**

| **Eating to stay healthy (YES)** | OR (95% CI) |
| --- | --- |
| Omnivorous diet (44594; 47.4% of this group) | 1 |
| Pesco-vegetarian diet^†^ (499; 55.5% of this group) | 1.79 (1.44-2.23) |
| Lacto-ovo-vegetarian / vegan diet^‡^ (369; 54.3% of this group) | 1.40 (1.07-1.82) |
| **Eating to stay healthy (NO)** |  |
| Omnivorous diet (49490; 52.6% of this group) | 1 |
| Pesco-vegetarian diet^†^ (400; 44.5% of this group) | 1.83 (1.45-2.30) |
| Lacto-ovo-vegetarian / vegan diet^‡^ (311; 45.7% of this group) | 3.00 (2.36-3.80) |

^†^ not eating meat or poultry

^‡^ not eating meat, poultry or fish (lacto-ovo-vegetarian and vegan diets were combined because of the low number of participants with vegan diet in the stratification analyses)
